# Squamous cell anal cancer: Management and therapeutic options

**DOI:** 10.1016/j.amsu.2020.04.016

**Published:** 2020-05-12

**Authors:** Beatrice Pessia, Lucia Romano, Antonio Giuliani, Gianni Lazzarin, Francesco Carlei, Mario Schietroma

**Affiliations:** Department of Surgery, Department of Applied Clinical Science and Biotechnology, University of L'Aquila, L'Aquila, Italy

**Keywords:** Anal cancer, Squamous-cell carcinoma, Chemoradiotherapy, Immunotherapy, Abdominoperineal amputation, Mitomycin C, 5-Fluorouracil

## Abstract

The incidence of anal cancer has increased during the second half of the 20th century, with an incidence rate over 2.9% greater than in the decade of 1992–2001. Yet, it still constitutes a small percentage, about 4%, of all anorectal tumours. Its risk factors are human papillomavirus infection, a history of sexually transmitted diseases, a history of vulvar or cervical carcinoma, immunosuppression related to human immunodeficiency virus infection or after organ transplantation, haematological or immunological disorders, and smoking. The most frequent symptom is rectal bleeding (45%), followed by anal pain, and sensation of a rectal mass. The diagnosis requires clinical examination, palpation of the inguinal lymph nodes, high resolution anoscopy followed by fine-needle aspiration biopsy or core biopsy. Subsequent histologic diagnosis is necessary, as well as computed tomography or magnetic resonance imaging evaluation of the pelvic lymph nodes. Since 1980, patients with a diagnosis of anal cancer have shown a significant improvement in survival. In Europe during the years 1983–1994, 1-year survival increased from 78% to 81%, and the improvement over 5 years was between 48% and 54%. Prior to 1974, patients with invasive cancer were routinely scheduled for abdominoperineal amputation, after which it was demonstrated that treatment with 5-fluorouracil and radiotherapy associated with mitomycin or capecitabine could be adequate to treat the tumour without surgery. Today, numerous studies have confirmed that combined multimodal treatment is effective and sufficient.

## Highlights

• The incidence of anal cancer has increased during the second half of the 20th century.
• The diagnosis requires clinical examination, high resolution anoscopy and fine-needle aspiration biopsy or core biopsy.
• Histologic diagnosis is necessary.
• Since 1980, patients with a diagnosis of anal cancer have shown a significant improvement in survival.
• In Europe during the years 1983-1994, 1-year survival increased from 78% to 81%.
• Today, numerous studies have confirmed that combined multimodal treatment is effective and sufficient.

## 1 Epidemiology of anal cancer (AC)

In the United States, about 8200 new cases of squamous cell carcinoma of the anus were reported in 2017, accounting for approximately 2.6% of reported tumours of the digestive system and being responsible for 1100 deaths. Compared to the cases from the decade 1992–2001, the incidence has increased considerably (over 2.9%), about 1.9 times for men and 1.5 times for women. The European annual incidence currently ranges from 3 per 100,000 for men in Geneva, Switzerland to less than 1 per 100,000 for both sexes in England and Holland. In general, the incidence of AC in women is about twice that of men and the tumours of the anal region constitute about 4% of all anorectal tumours. Another important characteristic of this disease and its rise is that its epidemiology has changed considerably over the second half of the 20th century [1,2].

## 2 Risk factors of AC

AC risk factors include human papillomavirus (HPV) infection, history of sexually transmitted diseases, history of vulvar or cervical carcinoma, immunosuppression related to human immunodeficiency virus (HIV) or subsequent to organ transplantation, haematological or immunological disorders, and smoking.

The risk of developing AC is remarkably high in HPV-positive individuals, especially among those with HPV serotypes 16 and 18. Indeed, a recent systematic review showed that 72% of patients with invasive carcinoma tested positive for HPV DNA, and the United States Control and Prevention Center decreed in 2012 that between 86% and 97% of the reported ACs were attributable to HPV infection.

Pharmacological or HIV-linked immunosuppression promotes anal HPV infection. In fact, the annual incidence of anal cancer reached 103 cases per 100,000 person-years in some HIV populations after 1996 from 15 cases per 100,000 person-years before 1996. Until the late 2000s, multiple population-based studies have shown that anal cancer incidence has not declined in people living with HIV, despite the enormous improvements in ART and treatment coverage and the consequent improvement of immune function [3]. Among Japanese HIV-infected patients, approximately two-thirds of MSM, one-fifth of heterosexual men, and one-fifth of women have anal oncogenic HPV infection. Younger age, MSM, ≥2 STIs, and immunosuppression confer a higher risk of infection with oncogenic HPV and multiple oncogenic types [4]. Suppression of the immune system by the use of immunosuppressive drugs or HIV infection likely facilitates persistence of HPV infection of the anal region. Studies have shown that people living with HIV (PLWH) have an approximately 15- to 35-fold increased likelihood of being diagnosed with anal cancer compared with the general population. In PLWH, the standardized incidence rate of anal carcinoma per 100,000 person-years in the United States, estimated to be 19.0 in 1992 through 1995, increased to 78.2 during 2000 through 2003 [5,6]. The same prevalence is in Asian country [7]. This result likely reflects both the survival benefits of modern antiretroviral therapy (ART) and the lack of an impact of ART on the progression of anal cancer precursors.

## 3 Survival

From the European Cancer Registry Based Study on Survival and Care of Cancer (known as “EUROCARE”) [8] study, the overall survival in AC is measurable by data in the Cancer Registries of 22 European states. Survival assessment carried out for the 5386 adults in the Registry with the diagnosis of AC during the period 1983–1994 and followed up until 1999 yielded the following results. Relative survival for these adults of 90% at 1 year, 60% at 3 years and 53% at 5 years, with a small but significant difference between the women and men (55% and 50%, respectively). The 5-year relative survival was also found to decrease with age, from 65% for the youngest (15–54 years) to 41% for the elderly (75 years and over). Analysis further back, to 1980, showed a significant improvement in survival. In Europe during the years 1983–1994, the 1-year survival increased from 78% to 81%, and the 5-year survival increased from 48% to 54 [8].

## 4 Precancerous lesions of AC

The anatomopathological characteristics of AC are similar to those of cervical and vulvar lesions and the classification is the same.

### 4.1 European classification (Fig. 1) [9]

• Anal intraepithelial neoplasia (AIN) grade I: Cellular and nuclear abnormalities are confined to the lower 1/3 of the epithelium;
• AIN grade II: Cellular and nuclear abnormalities affect 2/3 of the epithelium;
• AIN grade III: Cellular and nuclear anomalies affect the full thickness of the epithelium.

### 4.2 American classification

• Atypical squamous cells of undetermined significance (referred to as “ASCUS”);
• Low-grade intraepithelial lesion (referred to as “LSIL”);
• High-grade intraepithelial lesion (referred to as “HSIL”).

AIN I and AIN II correspond to LSIL, and AIN III corresponds to HSIL. The real incidence of AIN is not known. Although, alterations attributable to AIN have been detected in 0.2%–4.4% of minor surgery of the anal edge [10].

The aetiologies of AIN and squamous AC seem to be the same, with HPV being involved especially. In particular, the HPV subtype 16 has been identified in 56%–96% of patients with squamous cell carcinoma or with AIN III lesions. It is also possible that HPV has a synergistic action with other viruses (*e.g*., HIV, herpes simplex virus, cytomegalovirus, Epstein-Barr virus) and with immunocompromised conditions.

The rate of progression from an AIN lesion to invasive carcinoma is not completely clear, but a high incidence of AIN III has been reported in patients with AC (80%). In the study by Zaccarini et al. [10], out of 32 patients with AIN III, 5 (15.5%) developed an AC in about 18 mo (range: 0.5–2 years). Moreover, the progression from AIN I to AIN III has been reported, not only for perianal skin but also for the transition epithelium.

Anoscopy and acetic acid 5% are used for the diagnosis. Anal cytology is also suggested for screening to detect dysplasia, as is high-resolution anoscopy. In general, however, suspicious areas must be biopsied.

An ongoing challenge to treatment is the absence of data demonstrating efficacy of the therapy [11]. In AIN I and AIN II, clinical observation is suggested, unless there are evident or ulcerated lesions. In the case of AIN III, there is the risk of progression to carcinoma, but there is also high morbidity connected with surgery for patients who are frequently immunocompromised. In the case of evident lesions, anal mucosa removal has to be done, applying plastic surgery techniques [10]; no case of spontaneous regression of AIN has been reported [12].

Screening for AIN detection in HIV patients is controversial. A randomized trial [13] showed no benefit from screening programs, as concerning mortality reduction; however, some guidelines recommend screening with rectal examination in HIV-positive subjects.

The American Society of Colon and Rectum Surgeons recommends excision with electrocautery, and this procedure appears to be more effective than topical therapy with imiquimod and fluorouracil (FU) [14].

Moreover, the group of patients with perianal lesions appears to respond better to the topical treatment than does the group with intra-anal lesions [15].

## 5 Pathophysiology

The anal region is located between the anal canal and the anal margin. The anal margin itself includes the perianal skin, 5–6 cm up to the mucocutaneous junction. It is covered by epidermis, and not mucosa. The AC tumour can originate from the margin or from the anal canal [16], which serves as the basis for their classification into two categories.

In accordance with the latest edition of the World Health Organization classification system (edition IV), the histotype of AC is the squamous cell, with subtypes being variants with transitional cells, large keratinized cells, large nonkeratinized cells, or basaloid cells [17]. Squamous cell AC of the anal margin is more frequent than the well-differentiated cell variant or the large nonkeratinized cell variant [18].

The AC lymphatic drainage depends on the location of the tumour. Tumours of the anal margin and of the anal canal under the dentate line drain into the superficial lymph nodes. Tumours of the anal canal region proximal to the dentate line drain into the anorectal, perirectal, and paravertebral stations, and in some cases into the internal iliac lymph nodes. Many of the proximal tumours drain in the perirectal lymph nodes and into the inferior mesenteric lymph nodes. However, patients with AC are at high risk of metastasis in the inguinal lymph nodes, since lymphatic drainage is not limited to the distal or proximal compartment and there is a rich network of connection between the two.

## 6 Screening

The existence of an identified viral aetiological agent and the ability to detect pre-neoplastic lesions may allow the development of screening and prevention programmes. Vaccination of girls against oncogenic HPV is now being recommended for the prevention of cervical cancer, and a recent report indicated that up to 80% of anal cancers could also be avoided with prophylactic quadrivalent HPV vaccine (against HPV types 6, 11, 16 and 18). But currently vaccination has no role when SCCA is actually present [19].

Screening programmes using anal cytology and high-resolution anoscopy have been proposed for high-risk populations (MSM and HIV– women with a history of anal intercourse or other HPV-related anogenital malignancies) based on the achievements obtained in cervical cytology screening. However, no randomised control study has yet demonstrated the advantage of screening in these high-risk populations [[20], [21], [22]].

There is no international consensus on AC screening strategy. Pap is currently the most accepted screening test for high-grade intraepithelial neoplasia (HGAIN). The sensitivity of anal Pap ranges from 47 to 70% for the detection of AIN of any grade, but seems higher in haiv men have sex with men (HIV-MSM) [11,23,24] reaching 89.2% for HGAIN detection in a recent study by Burgos et al. [25] In our study, anal Pap alone detected only 19 of 27 (70%) cases of HGAIN, which is, however, more than SA or HPV16 genotyping alone (respectively, 7 and 14 cases) [[26], [27], [28]].

Pernot et al. affirmed that single screening strategies, anal Pap alone had a higher HGAIN detection yield than SA and HPV-16 genotyping. Among the dual combination strategies, anal Pap + HPV-16 and SA + anal Pap had detection yields similar to that of the complete strategy. However, even though SA decreased the number of HRA performed, it is likely that it might also affect participation or acceptance. In the perspective of self-sampling, anal Pap + HPV-16 genotyping might be the best strategy to increase screening acceptance and to identify HGAIN in HIV-MSM, although it would increase the need for HRA. However, the added value of screening using detection of HPV-16 combined with other selected HR-HPV remains to be assessed in further studies. In addition, extending HRA accessibility is a remaining challenge that needs to be addressed. Finally, these strategies should be evaluated in further studies in the context of low HRA accessibility and health economics systems, with regard to acceptability by the patients [29].

The ongoing ANCHOR study (NCT02135419), currently recruiting more than 5000 HIV patients with HGAIN, randomised into two arms: experimental arm (topical or ablative treatment of lesions) or monitoring every 6 months, will provide a better understanding of the natural history of HGAIN and relevance of treatment. If ANCHOR study demonstrates that AIN treatment significantly reduces the incidence of anal cancer, then screening program for anal cancer will become the standard of care for HIV-infected patients.

## 7 Management

### 7.1 Tumor presentation and staging

The most frequent symptom is rectal bleeding (45%), followed by anal pain and sensation of rectal mass [30,31]. Identification of the size of the tumour, through clinical examination, is the first step for staging, but biopsy is required for histological confirmation. The previous American Joint Committee on Cancer guidelines also classified AC into the anal margin and anal canal categories. However, many tumours of the perianal area extend into the canal and precancerous lesions are sometimes found in it. For these reasons, the same staging system is used for both categories.

The National Comprehensive Cancer Network (commonly known as NCCN) Guidelines [32] for management of AC are provided in Fig. 2.

**Fig. 1:**
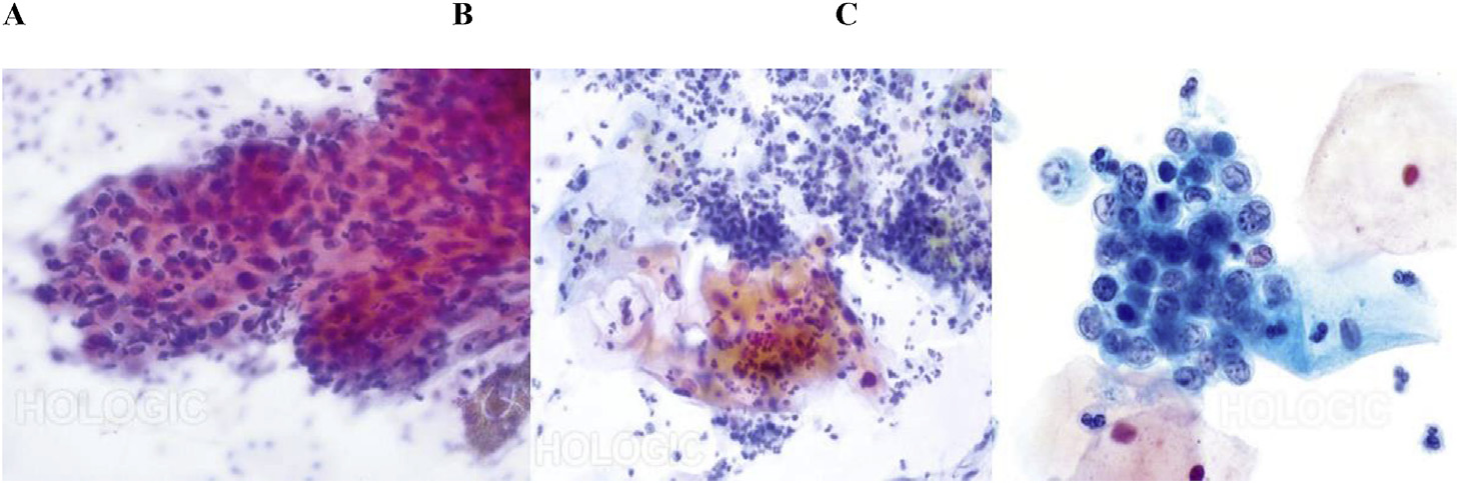
**Examples of European classification of precancerous lesions** [9]**.** A: AIN grade I: Cellular and nuclear abnormalities are confined to the lower 1/3 of the epithelium; B: AIN grade II: Cellular and nuclear abnormalities affect 2/3 of the epithelium; C: AIN grade III: Cellular and nuclear anomalies affect the full thickness of the epithelium. AIN: Anal intraepithelial neoplasia.

**Fig. 2:**
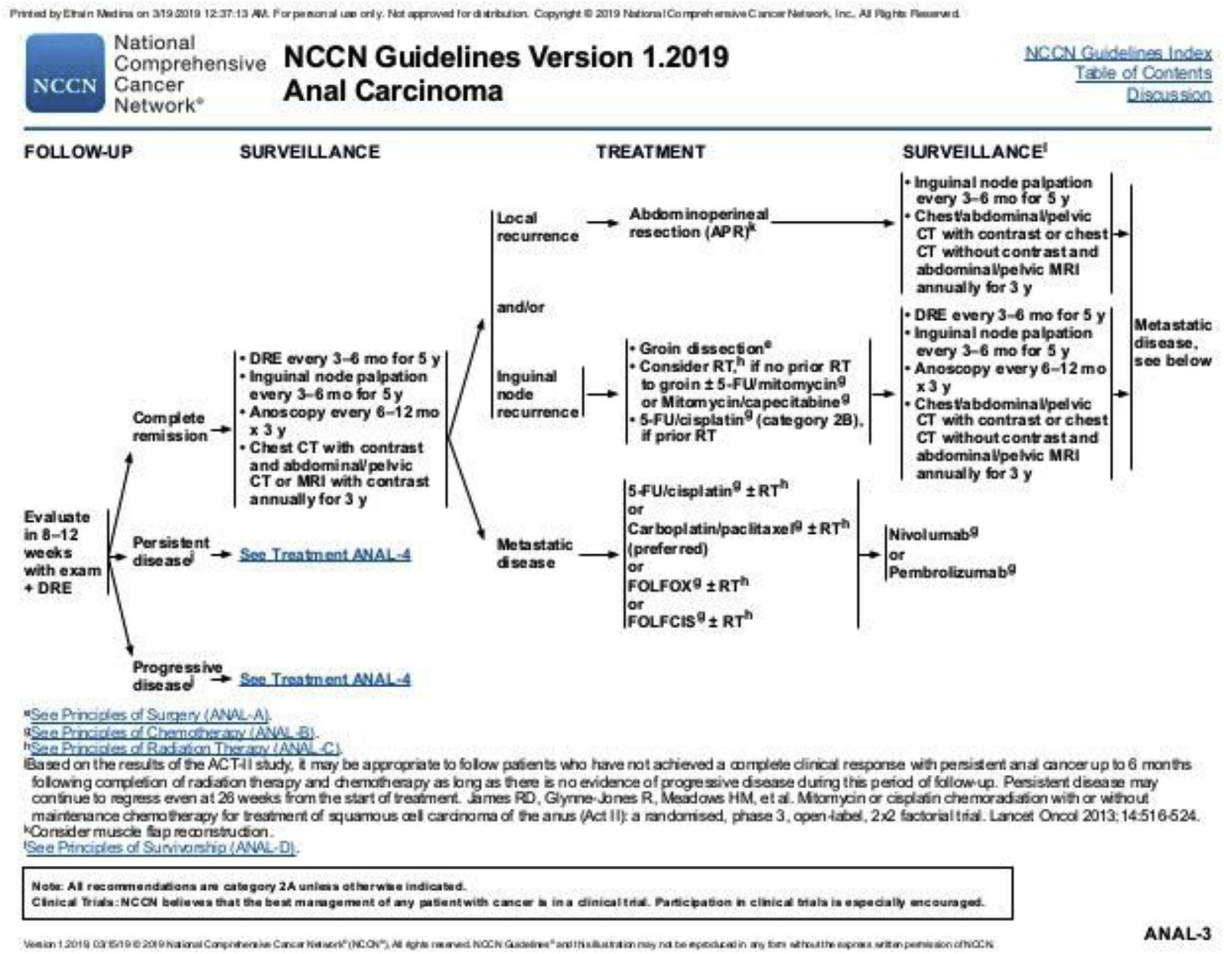
**NCCN guidelines for anal carcinoma (adopted from****Ref. [32]****).** NCCN: National comprehensive cancer network.

Diagnosis requires clinical examination, palpation of inguinal lymph nodes, and high-resolution anoscopy followed by fine-needle aspiration biopsy or core biopsy. After that, histologic diagnosis is necessary, as well as computed tomography (CT) or magnetic resonance imaging evaluation of the pelvic lymph nodes. Furthermore, CT scan of the abdomen is suggested, as it can show possible pelvic dissemination.

In patients at risk of AC, HPV tests are recommended, as is testing of levels of CD4-positive cells in HIV-positive patients. In women, gynaecological screening is required. Imaging by positron emission tomography-CT is helpful to verify the grade before treatment and is useful in the evaluation of pelvic lymph nodes in patients without CT evidence of the diagnosis [33].

The prognosis is related to the size of the tumour at the time of diagnosis, and to the presence of lymph node metastases. The 5-year survival rate is 89% in patients with early and local disease, falling to 12% in patients with distant metastases (Table 1). Male sex, positive lymph nodes, and tumour size greater than 5 cm are independent negative prognostic factors in terms of disease-free survival. Recent data in the literature indicate that infection with HPV serotype 16 is also related to a poor prognosis [33,34].

**Table 1:** **TNM score (adopted from****Ref. [32]****).** TNM: Tumour node metastasis.

DEFINITION OF TNM	
Primary Tumor (T)	
TX	Primary tumor cannot be assessed
T0	No evidence of primary tumor
Tis	Carcinoma *in situ* (Bowen's disease, high grade squamous intraepithelial lesion HSIL, anal intraepithelial neoplasia AIN II-III
T1	Tumor 2 cm or less in greatest dimension
T2	Tumor more than 2 cm but not more than 5 cm in greatest dimension
T3	Tumor more than 5 cm in greatest dimension
T4	Tumor of any size invades adjacent organ(s)*

*Note: Direct invasion of the rectal wall, perirectal skin, subcutaneous tissue or the sphincter muscle(s) is not classified as T4.	

**Regional Lymph Nodes (N)**	
NX	Regional lyph nodescannot be assessed
N0	No regional lyph nodes metastasis
N1	Metastasis in perirectal lyph node(s)
N2	Metastasis in unilateral internal iliac and/or inguinal lyph node(s)
N3	Metastasis in perirectal and inguinal lymph nodes and/or bilateral internal iliac and/or inguinal lymph nodes

**Distant Metastasis (M)**	
M0	No distant metastasis
M1	Distant metastasis

**ANATOMIC STAGE/PROGNOSTIC GROUPS**			
**Stage**	**T**	**N**	**M**
0	Tis	N0	M0
I	T1	N0	M0
II	T2	N0	M0
	T3	N0	M0
IIIA	T1	N1	M0
	T2	N1	M0
	T3	N1	M0
	T4	N0	M0
IIIB	T4	N1	M0
	Any T	N2	M0
	Any T	N3	M0
IV	Any T	Any N	M1

### 7.2 Initial management of local and locoregional disease

In the past, patients with invasive cancer were routinely scheduled for abdominoperineal amputation, but in any case, the 5-year mortality was very high (between 40% and 70%). In 1974, Nigro et al. [36] demonstrated that preoperative treatment with 5-FU and radiotherapy associated with mitomycin or capecitabine could be adequate to treat the tumour without surgery. To date, the standard of care for non-metastatic AC is CRT (chemotherapy plus Radiotherapy), the CT regiment usually is 5FU plus Mitomicin [36].

#### 7.2.1 Place of surgery

According to ESMO guidelines [37], NCCN [32], Saudi oncology society [38] and Japanese oncology group [39] from the 1970s surgery as the primary therapeutic option has generally been abandoned. Still today, smaller lesions (<2 cm in diameter), involving the anal margin and not poorly differentiated may be treated by primary surgery in the form of a local excision provided adequate margins (>5 mm) can be obtained without compromising sphincter function. Local excision has not been shown to be efficacious for small tumours in the anal canal and is contra-indicated. Primary abdomino-perineal excision (APE) was associated with local failure in up to half of cases, and 5-year survival rates in the region of 50%–70% were reported. Today, primary APE may be offered to patients previously irradiated in the pelvic region [[31], [37], [38], [39], [40]].

#### 7.2.2 Chemotherapy

A recent randomized clinical trial performed by the United Kingdom Co-ordinating Committee on Cancer Research demonstrated that chemoradiotherapy (CHRT) with 5-FU and mitomycin is much more effective in local disease control than radiotherapy alone, with the two groups of patients having the same percentage of disease-free survival at 3 years [[26], [27], [28]]. At the follow-up, the survival was 5.4 years in the radiotherapy group and 7.6 years in the CHRT group. Many other studies have confirmed the efficacy and safety of specific chemotherapeutic drugs in the CHRT regimen; in fact, the combination of 5-FU with mitomycin produced a low colostomy rate and high rate of disease-free survival at 4 years (9% *vs* 22% and 73% *vs* 51%, respectively) compared to the group treated only with 5-FU.

Capecitabine, which belongs to the class of fluoropyrimidines, is an oral prodrug that represents a valid alternative to 5-FU in the treatment of colonic and rectal cancer. As such, it has potential in treatment of AC as an alternative to 5-FU in chemotherapy regimens for cases of nonmetastatic cancer.

Meulendijkis et al. [25] reported their comparative study of 58 patients treated with capecitabine *versus* 57 patients treated with infusion of 5-FU, with radiotherapy and mitomycin for both groups. There were no significant differences found between the two groups for local response, 3-year locoregional control, 3-year overall survival, and 3-year colostomy-free survival. Goodman et al. [41] showed the same results; in addition, they demonstrated that hematologic toxicity of grades 3 and 4 was significantly reduced in patients treated with capecitabine.

The cisplatin used in the treatment of metastatic AC can be a substitute for mitomycin. In a recent study published in the *Lancet*, James et al. [42] had investigated more than 900 patients, randomized into two groups for 5-FU with mitomycin treatment or 5-FU with cisplatin treatment, both with two doses of 50.4 Gy radiotherapy. No differences were found at the 5-year follow-up, in terms of complete response rate, disease-free survival, and colostomy-free survival. A recent phase I trial of IMRT and concurrent chemotherapy using paclitaxel, capecitabine, and Mitomicin for AC reported that 33 of 38 patients (86.8%) achieved a complete clinical response at 26 weeks [43]. Interesting will be to wait for the results of the Russian Phase III Trial, which will end in 2021, comparing paclitaxel, capecitabine, and mitomicin combined with IMRT against the standard capecitabine and Mitomicin [44]. CRT is effective for early-stage cancers, but overall the disease fails to respond or relapses locally within 2 years for 20%–30% of patients [45].

#### 7.2.3 Radiotherapy

The optimal radiotherapeutic scheme for AC is still not standardized; however, collective data from the literature suggests using 40–50 Gy in patients with T *in situ* and 50–60 Gy for T1 stage patients [46]. Another study conducted with patients with T3 or T4 or N+, with radiotherapy greater than 54 Gy but less than 60, showed greater control of locoregional disease. No benefit was seen with higher doses, and to the detriment of high toxicity.

There is evidence in the literature that interrupted treatments due to radiotherapy-related toxicity compromise the efficacy of treatment. In the RTOG9208 phase II study, the AC patients administered a biweekly scheme had a higher locoregional recurrence risk and a lower rate of colostomy-free survival than the single-dose patients; although, the latter had increased rate of skin toxicity.

In contrast, the findings from other studies [[47], [48], [49], [50]] have shown benefit in terms of locoregional control of the disease, with reduced toxicity, if the CHRT protocol is delivered in short periods. For example, if the administration of 30 Gy in 3 wk produced anoproctitis and perianal dermatitis in one-third of the patients, this percentage doubled if the scheme was 60 Gy for 6 wk.

Radiotherapy-related toxicity is represented by an increase in defecatory urgencies, chronic perianal dermatitis, dyspareunia, and impotence. In many cases, the radiotherapy caused complications requiring a colostomy, such as anal ulcers, stenosis, and necrosis. A retrospective study on the data in the Surveillance, Epidemiology and End-Results (commonly known as SEER) registry showed a 3-fold increase in pelvic fractures for elderly women who received radiotherapy compared to those who did not. However, thanks to the introduction of new irradiation techniques, the relative toxicity has decreased, especially with intensity-modulated radiotherapy (IMRT).

Sakanaka et al. [51] demonstrated how simultaneous integrated boost intensity-modulated radiotherapy significantly reduced doses to the external genitals, bladder and intestine, providing better focused doses to the target and nodal-elective region. At the mean follow-up time of 46 mo, the locoregional control at 3 years and the overall survival rate were 88.9% and 100%, respectively. Acute toxicity was treated conservatively. All patients completed radiotherapy with brief interruptions (Fig. 1). Ultimately, the intensity-modulated radiotherapy showed less toxicity compared to conventional treatment, and good results on overall survival at 3 years.

#### 7.2.4 Anti-EGFR and biologic therapy

The inhibitor of the epidermal growth factor receptor (commonly known as EGFR) such as Cetuximab and Panitunumab and their antitumoral activity depends on the presence of nonmutated KRAS, the mutation of which is very rare in AC. Although chemotherapy for squamous carcinoma of the anal canal allows for preservation of the sphincter, it is associated with a high rate of locoregional recurrence in general. However, for HPV-positive oropharyngeal tumours, cetuximab can enhance the therapeutic effect of radiation therapy. In the E3205 phase II study conducted by the Eastern Cooperative Oncology Group of 2017, Garg et al. [52] hypothesized that the addition of cetuximab to a chemotherapy regimen would reduce local recurrence in patients with AC. Sixty-one patients received chemotherapy with cisplatin plus 5-FU and radiotherapy (from 45 Gy to 54 Gy) to the primary tumour and locoregional lymph nodes, augmented by eight weekly doses of cetuximab. The study aimed to obtain at least a 50% reduction in locoregional recurrence rates at 3 years, as compared to the standard chemotherapy scheme known to achieve 35% reduction. Unfortunately, grade 4 toxicity occurred in 32% of the patients receiving the experimental treatment, and 5% died from chemotherapy consequences. Ultimately, the survival rate without local recurrence at 3 years was 23%. Similar results have been obtained from phase II trial by the Grupo Español Multidisciplinar en Cancer Digestivo (GEMCAD) with panitumumab, Mimotmicin, FU, and radiotherapy, 33 of 36 patients (92%) developed grade 3 or 4 adverse events, and outcomes were similarly poor [53]. The researchers concluded that, even though the addition of anti-EGFR agents associated with low rates of locoregional recurrences compared to the standard scheme, the toxicity was substantial.

The intuition to be able to act on related hpv immunogenicity of cancer anal has conducted research groups to test the effectiveness of anti-pd1, such as nivolumab and prembrolizumab [54]. Nivolumab may impair the ability of residual but damaged tumor cells to grow and spread following crt. Patients with HIV are eligible if their CD4 count is greater than 200 cells/mm3. Primary end point is os, secondary end-point is response rate, toxicity, and colostomy-free survival. The phase 1B/II trial of pembrolizumab plus IMRT in stage III/IV carcinoma of anus (CORINTH) is a multicenter trial with a single arm. Patients will be recruited into 3 successive cohorts followed by an expansion of the final cohort. for each cohort the first dose of pembrolizumab will be given at an earlier time point during the chemo-radiation (crt). Safety and tolerability and response rate will be assessed [55].

## 8 Treatment of metastatic AC

Less than 20% of all patients with SCCA will present with surgically unresectable or metastatic disease, with an estimated 5-year OS of 30%.58 Prior to 2017, no trials dedicated to the treatment of metastatic anal cancer had been explored, with data limited to retrospective analyses [56].

Until 2017, the recommended treatment was the combination of cisplatin + 5FU, with response rates of 50–60% and median survival of 12 months. Therefore, Kim et al. [57] publishes on *Lancet Oncology* in 2018 the results obtained in a patient with relapsing metastatic or unresectable disease treated with Docetaxel (standard or modified dose) Cisplatin 5FU. the rationale is based that Docetaxel is a powerful microtubule-stabilizing agent, with an antitumoral activity that leads to the mitosis arrest and cell death. It has been proposed that a loss of normal p53 function sensitizes the tumour to taxane chemotherapy by increasing G2/M arrest and apoptosis.

Since the association between AC and HPV infection is particularly strong and the E6 oncoprotein encoded by HPV types 16 and 18 induces p53 degradation, it has been hypothesized that AC could be sensitive to chemotherapy containing taxanes [58]. The multicentre study Epitopes-HPV02 that recruited patients from 25 academic hospitals. The study satisfied the primary end point with 47% of patients free from disease progression at 12 months, PFS of 11 in the modified scheme. the objective response rate was 86% with 44% CR, 70% of patients developed grade 3–4 toxicity. In light of these data, DCF represents the first line in cases of metastatic AC in patients EGOS 0–1.

In 2001, Hainsworth et al. [59] published their findings from a phase II trial study. A total of 60 patients were included between February 1995 and March 1999, including 12 (20%) who had received a previous chemotherapy regimen and 48 (80%) who had not received any previous treatment. All patients received the following regimen: paclitaxel at 200 mg/m2 and intravenous infusion for 1 h on d 1 and d 22; carboplatin under the concentration-time curve 6.0 intravenously on d 1 and d 22; 5-FU at 225 mg/m2 per d by 24-hr continuous intravenous infusion from d 1 through d 35. The treatment courses were repeated at intervals of 6 wk; responding patients continued the treatment for up to four courses (24 wk). At the end, 65% had objective responses to this regimen, while 25% had a complete response. Twelve patients (22%) remained disease-free from 7 mo to 63 mo (median: 35 mo) after the conclusion of therapy. Complete responses were observed in squamous carcinomas from various primary sites, including head and neck, oesophagus, cervix, vagina, and anus. The most frequent toxicities of grades III and IV observed with this regimen of three drugs included leukopenia (48%), diarrhea (17%), mucositis (28%) and proctitis-related events (13%).

In according to NCCN 2020 [32] paclitaxel plus carboplatin has benne noted as the preferred regimen for the first line treatment of metastatic anal cancer, based on result from phase II international Multicentric Inter-AACT study. In this study 91 pts were randomized to either carboplatin plus paclitaxel or cisplatin plus 5FU. While response rates were similar between carboplatinun plus paclitaxel and cisplatin plus %FU (59.0% and 57.1% respectively), carboplatin plus paclitaxel showed lower toxicity compared to cisplatin + 5FU, (71% vs 76% grade >3 toxicity and 36---5 vs 62-5 serious adverse events.). Median PFS and OS were 8.1 months and 20 months for carboplatin plus paclitaxel, and 5.7 months and 12.3 months for cisplatin plus 5FU (HR for OS 2.0; p = 0.014). Despite the limited data, FOLFCIS regimend could be standard option. A retrospective study of 53 patients with advances AC show 48% for the response rate, PFS was 7.1 months and OS were 22,1 months [60].

## 9 New frontiers: immunotherapy

Human tumours have numerous genetic and epigenetic alterations, generating neo-antigens potentially recognizable by the immune system [61]. Although an endogenous immune response to cancer is observed in preclinical models and in patients, this response is ineffective because tumours develop multiple resistance mechanisms. Furthermore, the tumours can use different distinct pathways to actively avoid immune destruction; these include endogenous immunogenic ‘controls’ that normally stop immune responses after antigen activation. These observations have motivated intensive efforts to develop immunotherapeutic approaches for cancer, one of the first of which being immune control pathway inhibitors such as the anti-CTLA-4 antibody (ipilimumab) for the treatment of patients with advanced melanoma.

Programmed death receptor-1 (PD-1) is a receptor involved in the programmed cell death signalling pathway. Serving as a key receptor of immune control, it is expressed by activated T cells and mediates immunosuppression. PD-1 works mainly in peripheral tissues, where T cells can interact with the immunosuppressive ligand-1 immune cell control molecule of programmed death (PD-L1, B7-H1) and PD-L2 (B7-DC), both of which are expressed by tumour cells, stromal cells or both [[16], [17], [18],^33^]. Inhibition of the interaction between PD-1 and PD-L1 can improve T cell responses *in vitro* and mediate preclinical antitumor activity. In a dose escalation study, the anti-PD-1 monoclonal antibody BMS-936558 (also known as MDX-1106 and ONO-4538) was administered as a single dose to 39 patients with advanced solid tumours [62]. Although this pilot study is currently underway, the findings include a favourable safety profile and preliminary clinical activity for multiple doses involving patients with different tumours.

Many tumours evade immune surveillance and destruction through the up-regulation of PD-L1. The interaction between the PD-1, expressed in tumour infiltrating T cells, and its PD-L1 ligand leads to functional inactivation of T cells; this mechanism is known as adaptive immune resistance [63]. Monoclonal antibodies against PD-1 and PD-L1, including pembrolizumab, nivolumab and atezolizumab, have shown antitumor activity in a different set of tumour types. A correlation between the PD-L1 expression in pretreatment and the response to anti-PD-1 therapy has also been reported in different tumour types.

### 9.1 Pembrolizumab

The KEYNOTE-028 study evaluated pembrolizumab monotherapy in 20 different types of advanced or recurrent PD-L1-positive tumours with significant medical need. The results of the KEYNOTE-028 AC cohort are detailed herein [57].

**Fig. 3:**
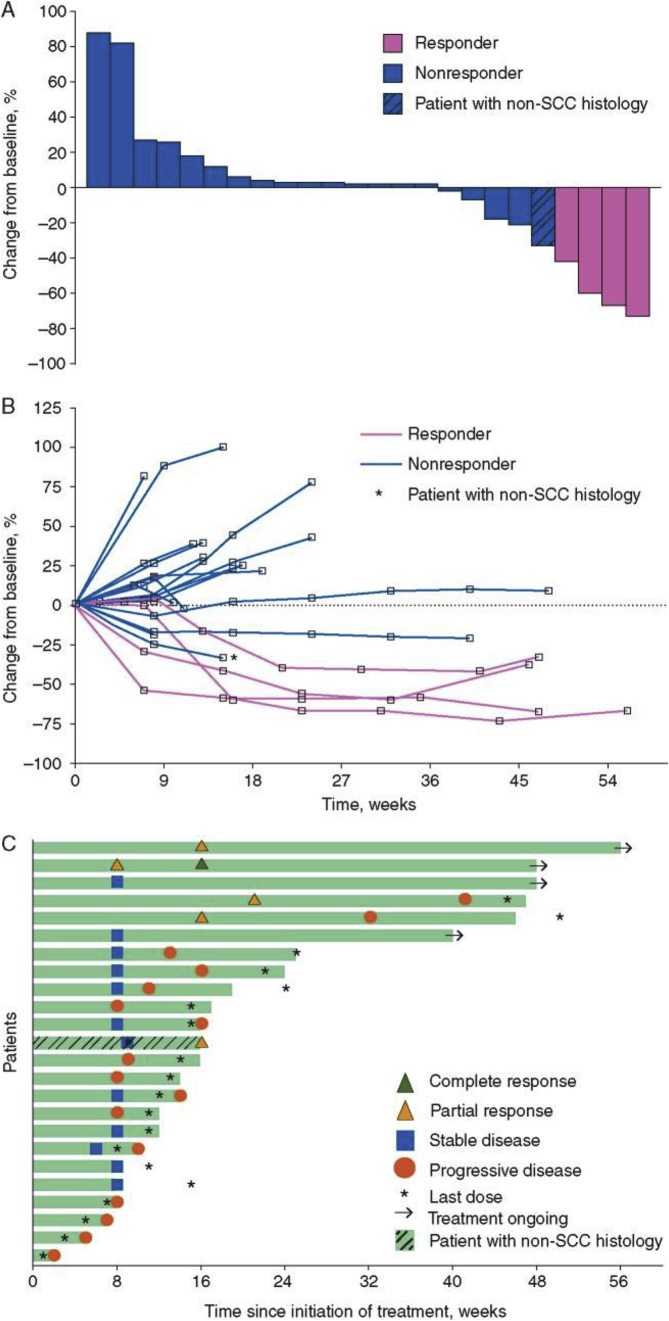
**Safety and antitumor activity of the anti-PD-1 antibody pembrolizumab in patients with recurrent carcinoma of the anal canal** (**adopted from****Ref. [91]****).** A: Maximum change from baseline in tumour size. Includes patients with ≥1 postbaseline tumour assessment (*n* = 24). Responders were defined as patients having confirmed complete response or partial response per RECIST v1.1 by investigator review; B: Longitudinal change from baseline in tumour size. Includes patients with ≥1 postbaseline tumour assessment (*n* = 24). Responders were defined as patients having confirmed complete response or partial response per RECIST v1.1 by investigator review; C: Treatment exposure and response duration. The length of each bar represents the time to the last radiographic assessment. Both confirmed and unconfirmed responses were defined per RECIST v1.1 by investigator review.

Patients with PD-L1-positive tumours (≥1%) received pembrolizumab at 10 mg/kg intravenously every 2 wk for up to 2 years or until confirmed progression of unacceptable toxicity. The response was evaluated every 8 wk for the first 6 mo and every 12 wk thereafter for response assessment criteria in solid tumours. The primary end-points were safety and overall response rate; the secondary end-points included progression-free survival, overall survival, and duration of response. The data interruption date was July 1, 2015. Of the 43 patients with advanced AC assessable for PD-L1 expression, 32 (74%) had positive PD-L1 tumours as assessed by the 22C3 prototype test, of which 25 enrolled between April and September 2014. Sixteen patients (64%) experienced treatment-related adverse events; the most common were diarrhea and fatigue (4 patients, 16%) and nausea (3 patients, 12%). No deaths occurred nor discontinuances of treatment since the date of interruption. Among the 24 patients with histology of squamous cell carcinoma, 4 patients had confirmed partial response, for an overall response rate of 17% (95% confidence interval: 5%–37%) and 10 (42%) had stable disease, for a disease control rate of 58% [64] (Fig. 3). An additional patient with nonsquamous histology had confirmed stable disease.

In this population of patients with advanced PD-L1-positive squamous cell carcinoma, pembrolizumab demonstrated a manageable safety profile and encouraged antitumor activity. These data further support the study of pembrolizumab for this patient population.

### 9.2 Nivolumab

More than 90% of ACs are linked to a previous HPV infection. Preventive HPV vaccination is under-studied but, although many adolescents are vaccinated, the incidence of AC is growing. The viral HPV E6 and E7 oncoproteins are implicated in the neoplastic transformation of squamous cells of the anal canal into invasive carcinoma. The oncoproteins are immunogenic and can trigger an immune antitumor response for the recruitment of tumour infiltrating lymphocytes [[65], [66], [67]].

Cancer cells are known to express PD-L1; its PD-1 inhibitor receptor, expressed on the surface of T cells, when bound, down-regulates T cell activation and hinders the local antitumor immune response [68,69]. Nivolumab is a humanized monoclonal antibody against PD-1 that interferes with this interaction, allowing the T cell to be cytotoxic. This drug has demonstrated activity as an advanced monotherapy in solid tumours, such as head and neck cancer, melanoma, non-small cell lung cancer and renal cell carcinoma.

**Fig. 4:**
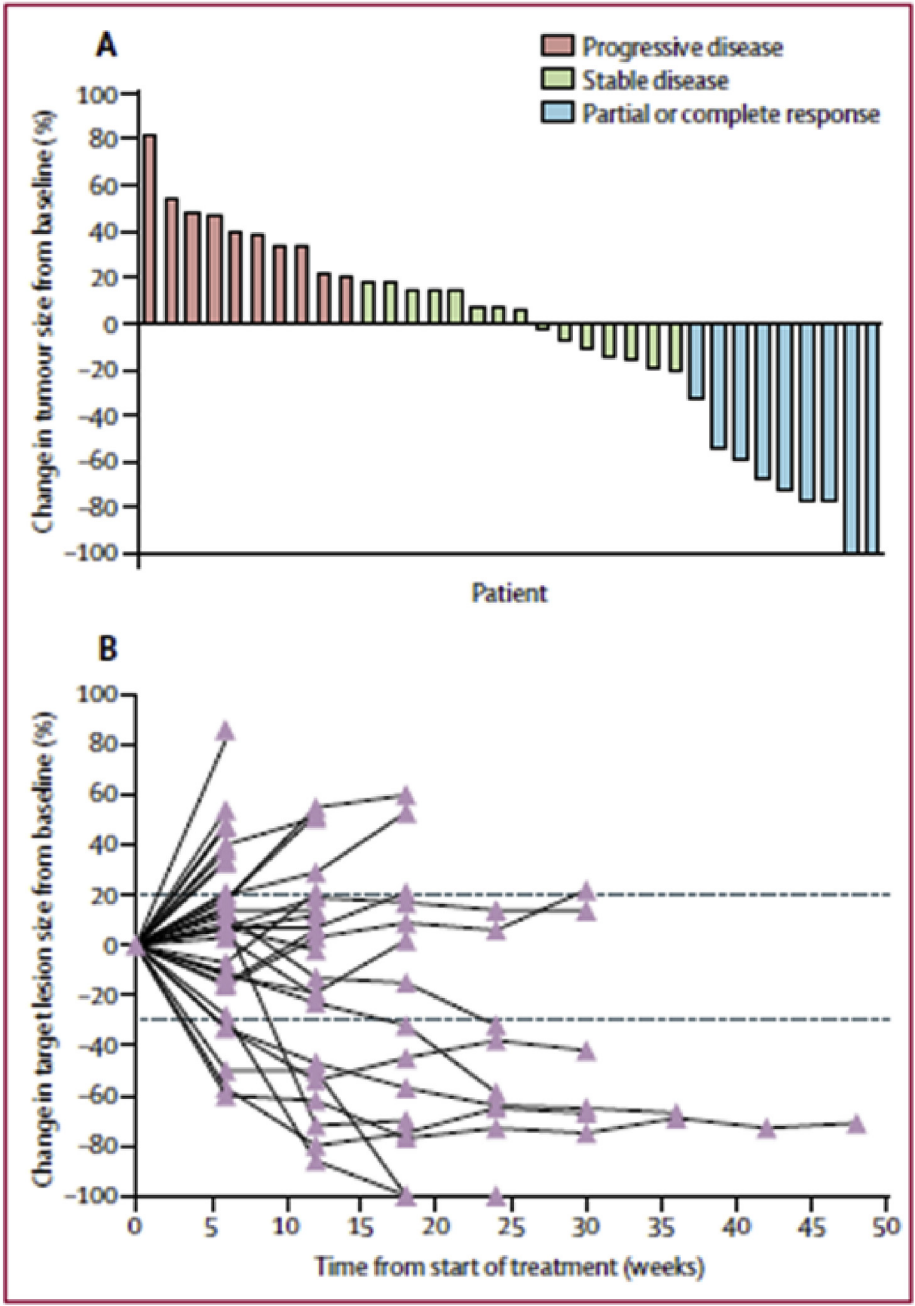
**Tumour response to nivolumab in 34 assessable patient**s **(adopted from****Ref. [92]****).** A: Waterfall plot; B: Duration of response.

In a recent multicentre study published in the *Lancet*, Van Morris et al. [65] reported their study of the efficacy of nivolumab in patients with metastatic AC (Fig. 4). A total of 37 patients received treatment with cisplatin-nivolumab after failure of standard therapy. The administration was intravenous, every 2 wk, at a dose of 3 mg/kg. The toxicity of the drug was tested at the first administration and after 2 wk. In any case in which the toxicity was greater than 2° or such as to require doses of corticosteroids, the administration was interrupted and not resumed until the degree of toxicity returned to 1. The primary end-point was regression of disease (demonstrated by radiological investigations). The secondary end-point was disease-free progression (defined as the time elapsed between the beginning of the treatment and the tumour progression or death).

The average follow-up time in this study was 101 mo, and patients received an average of six doses of nivolumab. Restaging was not possible in 3 patients. In 9 patients a response was obtained, including 7 partial and 2 complete. A durable response was obtained in 8 of the 9 patients, with a median duration of 5–8 mo. The mean reduction in the target lesion was 70%. Adverse reactions were anaemia, asthenia, hypothyroidism (all three being resolved after corticosteroid therapy), rash, and pneumonia. Nivolumab resulted in an objective response rate of 24% for the patients with metastatic squamous AC.

All patients had previously received treatment for incurable, metastatic AC. Although the progression-free median survival was 4.1 mo, the longest duration of treatment was almost 1 year. Interpretation of the data showed that, in the analysis of pretreatment biopsies, an association existed between responses to treatment and the presence of inflammatory infiltrate inside the tumour before the administration of nivolumab. Responding tumours had more activated T cells than the nonresponding. A cytometric analysis showed that there was a greater expression of PD-1 and PD-1L in the tumours that responded to treatment. To increase the efficacy of immunotherapy, the authors stated plans to combine nivolumab with ipilimumab in future studies. This approach has already had some success in the treatment of melanoma, based on the combination with an antiangiogenic drug or an anti-inflammatory agent (*e.g*., aspirin) that reduces the inflammatory infiltrate present in patients with AC to positively influence the response to nivolumab.

PD-1 inhibitors act through a T cell-dependent mechanism, decreasing circulating CD8 T cells; the underlying mechanism aligns with the hypothesis that lack of tumour-infiltrating lymphocytes caused by inflammation associated with HPV infection could cause treatment failure. NF-κB, interleukin 1β and interleukin 6 are often overexpressed in AC, which could also explain resistance to PD-1 inhibitors. Positive results with EGFR inhibitors for AC could suggest heterogeneity of the altered treatment pathways and how this molecular heterogeneity could cause different immunological states.

A further topic for future study is the effects of immunotherapy applied early in the pathological course of AC. Radiation therapy appears to work synergistically with anti-PD-1 therapy, so a combination of nivolumab and radiotherapy might be worthy of new studies [70]. ADXS11-001 is a live attenuated Listeria monocytogenes bacterium bioengineered to secrete a HPV-16-E7 fusion protein, which then targets HPV-transformed cells. The resulting ADXS11-001/HPV E7 antigen provokes immune cells to attach to AC cells expressing HPV E7. A phase II first-in-human study to assess the efficacy and safety of ADXS11-001 in patients with previously treated advanced AC was completed. 72 The primary endpoint was a 6-month PFS of 20%. Thirty-six patients were enrolled. Overall, the ORR was only 3.4% and the 6-month PFS (15.5%) failed to meet its primary endpoint [71,72].

## 10 Treatment of progressive or recidivant AC

Despite the efficacy of CHRT as a primary treatment of AC, the locoregional failure rate is between 10% and 30%. Many of the reported cases of primary treatment failure have been advanced-stage tumours with high-T and positive lymph nodes. Evidence of disease progression can be obtained by digital exploration, followed by biopsy, or through such instrumental exams as CT or positron emission tomography-CT. Patients with locally progressive disease are candidates for radical surgery with abdominoperineal amputation (APR) and definitive colostomy.

In a multicentre retrospective cohort study looked at the causes-specific colostomy rates in 253 patients with anal cancer, who were treated Rt or CTR. The 5-year cumulative incidence were 26% (95% CI, 2%–32%), and 8% (95% CI, 5%–12%) respectively. Large tumor size (>6 cm) was a risk factors dr tumor specific colostomy, while local excision prior to RT was a risk for therapy-specific colostomy [73,74].

Wright et al. [75] performed a prospective study to assess the risk of local recurrence in patients treated with surgery associated with high-dose intraoperative radiotherapy. Of 14 patients, 8 underwent abdominoperineal amputation with or without sacrectomy, 2 underwent anterior rectal resection, and 4 underwent pelvic exenteration. The average radiation dose was 1500 Gy, and the average follow-up was 17 mo. Recurrence was found in 11 cases. The authors concluded that rescue surgery with high-dose intraoperative radiotherapy did not appear to be associated with locoregional control or a survival benefit in these patients. Thus, the addition of high-dose intraoperative radiotherapy for rescue surgery is insufficient to compensate for positive surgical margins.

Hagemans JAW et al. analyzed oncological and surgical outcomes of our 30-year experience with salvage APR for AC after failed CRT. Forty-seven patients underwent salvage APR for either persistent (n = 24) or recurrent SCC (n = 23). Median OS was 47 months [95% confidence interval (CI) 10.0–84.0 months] and 5-year survival was 41.6%, which did not differ significantly between persistent or recurrent disease (p = 0.551), 5-year local recurrence rate was 44.7%. There was no difference in survival between persistent or recurrent anal SCC. The study confirms the benefit of salvage APR for persistent or recurrent anal SCC after failure of primary treatment with CRT. Surgical treatment of re-recurrence after salvage APR, however, does not appear to be useful [76].

Fields et al. investigated survival in patients with AC who fail first-line treatment. There were 256 patients in the early salvage group who underwent abdominoperineal resection (APR) within 6 months of completing chemoradiotherapy and 181 patients in the late salvage group who had APR 6 months or more after completion of chemoradiotherapy. Both groups of patients had similar tumor size (45 vs 50 mm; P = 0.07) and rate of positive margins (21.5% vs 15.6%; P = 0.13). The OS in patients undergoing early vs late salvage APR after failure of chemoradiotherapy is similar. As a result, the authors concluded that patients with persistent disease should be offered surgery just as readily as those with recurrent disease [77]. Finally, preoperatory imaging should be used to assist in patient selection, to identify those patients in whom negative margins can be obtained and to determine an appropriate rescue surgery. After abdominoperineal amputation, patients should be re-evaluated every 3–6 mo for 5 years, with clinical evaluation of the lymph nodes or their evaluation with pelvic CT.

## 11 Toxicity and complications induced by therapy

Prevention and management of both acute and chronic gastrointestinal (GI) side effects of pelvic RT have been a focus of recent research and reviews [[78], [79], [80]]. The most common acute toxicity, diarrhea, is typically managed with a combination of antidiarrheal agents (ie. loperamide and diphenoxylate/atropine), bulking agents, dietary modification, hydration and medication management (to minimize or substitute those medications that may promote diarrhea). A Cochrane review recently assess the role of dietary modification in minimizing acute diarrhea during pelvic RT and found that fat-restricted and fiber-supplemented diets may ameliorate diarrhea.

Radiation-induced late complications usually appear 6–12 months after treatment and affect 5–11% of patients [35,81]. Typical manifestations include obstructions, compromised motility, perforations, malabsorption and fistulas. Complications derive from the progressive evolution towards fibrosis and the chronic ischemia that affect and subvert the treated tissue. These processes may be asymptomatic and undiagnosed, inevitably making the involved structures more vulnerable to additional insults [49,82]. Rectal bleeding is generally managed initially with endoscopic evaluation followed by bowel habit optimization and medical therapy, include sucralfate enemas and oral metronidazole. Several bleeding by chronic radiation proctitis has sometimes been treated with hyperbaric oxygen therapy [83]. However, a double-blind RCT evaluating hyperbaric oxygen for pelvic RT patients with late GI toxicities demonstrated no benefit in patients with chronic GI toxicities after pelvic RT [84].

After APR for recurrent or persistent AC one of the major postoperative complications is perineal wound healing which often results in a prolonged hospital stay or even the need for surgical re-intervention [[85], [86], [87]]. Literature data reported a reduction of perineal wound complications if perineal reconstruction was achieved using a vertical rectus abdominis myocutaneous (VRAM) flap verus primary closure [88,89].

These data have been confirmed by Hartd et al., reviewing the outcomes of salvage surgery and perineal wound healing with or without a vertical rectus abdominis myocutaneous (VRAM) flap in a single institution over a 6-year period. One hundred twenty-four patients with AC were enrolled with a 5-year overall survival of 79%. Seventeen patients required (salvage) APR for recurrent (n = 8), persistent (n = 7), or primary anal carcinoma (n = 2). Median duration until completion of perineal wound healing was shorter in the VRAM group vs primary closure (17 vs. 24.5 weeks; p = 0.0541).

They also comparing surgical outcomes after abdominoperineal resection and VRAM for anal versus rectal cancer, we found a lower rate of perineal wound complications in the rectal cancer cohort. Moreover, there was a strong tendency towards a shorter duration of perineal wound healing in rectal cancer patients (8 versus 17 weeks), although this was not statistically significant. One reason for this remarkable difference could be the higher radiation dose to the pelvic region (median 54Gy, range 50.4–59.4Gy) in the anal cancer patients compared to the rectal cancer cohort who standardly received only 50.4 Gy [90].

## 12 Conclusion

AC is a rare cancer and accounts for approximately 4% of all cancers of the lower alimentary tract. The dominant aetiology is infection with human papilloma virus (HPV), which is the most common sexually transmitted disease. Vaccines directed against oncogenic HPV serotypes exist, and their utility for preventing anal neoplasia is under investigation. Additional risk factors for developing AC include HIV infection, anal receptive intercourse, smoking, and immunosuppression. Patients with known anal intraepithelial neoplasia (AIN) must be carefully screened with periodic digital rectal exam and anoscopy. The gold standard treatment for stage I-III disease is CRT (chemotherapy plus Radiotherapy), the CT regiment usually is 5FU plus Mitomicin. Lesion with <2 cm in diameter, involving the anal margin and not poorly differentiated may be treated by primary surgery in the form of a local excision provided adequate margins (>5 mm) can be obtained without compromising sphincter function. APR up front it is not indicated for high local recurrence rate which is around 50%, but is reserved in case of recurrence or persistence of tumor. Metastatic disease id treated with systemic CT, where Taxol plus 5FU and cisplatin or carboplatin, is the first line regimen. New frontiers is opened by immunotherapy, such as PD-1/PD-L1 inhibitors (Nivolumag and Prembrolizumab). AC may be responsive to PD-1/PD-L1 inhibitors because they often have high PD-L1 expression. Although further studies of PD-1/PD-L1 inhibitors are warranted, the panel added nivolumab and pembrolizumab as preferred options for patients with metastatic anal cancer who have progressed on first-line chemotherapy. Morbidity is high, mostly owing to wound complications, and a flap reconstruction of the perineum represent a salvage choice.

## Supported and conflict-of-interest statement

The authors declare no dedicated source of funding and no conflicts of interest related to this publication.

## Provenance and peer review

Not commissioned, externally peer reviewed.
